# High 1RM Tests Reproducibility and Validity are not Dependent on Training Experience, Muscle Group Tested or Strength Level in Older Women

**DOI:** 10.3390/sports6040171

**Published:** 2018-12-11

**Authors:** Matheus Barbalho, Paulo Gentil, Rodolfo Raiol, Fabrício Boscolo Del Vecchio, Rodrigo Ramirez-Campillo, Victor Silveira Coswig

**Affiliations:** 1 Faculdade de Educação Física e Dança, Universidade Federal de Goiás, Goiânia 74690-900, Brazil; paulogentil@hotmail.com; 2 Centro de Ciências Biológicas e da Saúde, Centro Universitário do Pará, Belém 66040-020, Brazil; rodolforaiol@gmail.com; 3 Escola Superior de Educação Física, Universidade Federal de Pelotas, Pelotas 96055-630, Brazil; fabricioboscolo@gmail.com; 4 Laboratory of Human Performance, Quality of Life and Wellness Research Group, Department of Physical Activity Sciences, Universidad de Los Lagos, Osorno 1305, Chile; r.ramirez@ulagos.cl; 5 Faculdade de Educação Física, Universidade Federal do Pará, Castanhal 68746-630, Brazil; vcoswig@gmail.com

**Keywords:** resistance training, strength test, muscle strength, older women

## Abstract

Background: The maximal one-repetition test (1-RM) is widely used in scientific research; however, there are conflicting results regarding its reproducibility in elderly populations. The present study aimed to analyze the reproducibility of the test both before and after a 12-week training period by using the bench press and leg press 45° 1-RM tests in the elderly, taking into consideration the training experience and strength level of the women. Methods: Elderly women (n = 376; age, 68.5 ± 14.1 years; height, 162.7 ± 5.5 cm; body mass, 71.2 ± 16.0 kg) who underwent ≥3 months of resistance training performed an initial week of familiarization and a second week of testing and retest, with a 48–72 h interval. Results: The results showed that Kappa indices ranged from 0.93 to 0.95, and the intraclass correlation coefficients were 0.99 for both the lower and upper limbs. In addition, minimal detectable changes were found that ranged between 1 and 3%, which means that changes lower than 1 kg could be detected. Conclusion: The present study confirms that the 1-RM test has high reliability and reproducibility in the elderly, for both upper and lower limbs.

## 1. Introduction

Along the aging process, muscle strength loss has been related to a reduction in quality of life [1,2] and increases in mortality [3]. To counter this, as an alternative to raising strength levels, resistance training (RT) has been widely suggested [4,5,6,7]. In fact, although it is widely accepted that the practice of RT can induce health benefits, the outcome itself (in terms of strength levels) has been proposed as being even more important than practicing RT to reduce mortality [8]. In addition, low levels of strength are predictive of increased risk of falls [9] and physical disability [10]. Based on this evidence, the evaluation of training responses, as well as strength status, has become important to the elderly.

Among the tests used to evaluate maximal strength, the maximal repetition test (1-RM) is considered the gold standard [11] and is suggested in important guidelines for exercise testing and prescription, mainly because it is practical and safe [12]. This test is characterized by performing a repetition in a given RT exercise with the highest load possible [11] and has been widely used as a parameter for load RT prescription and manipulation [13]. In addition, epidemiological and population studies have consistently associated the performance in 1RM tests with many health parameters [4].

However, the use of the 1-RM tests in the elderly has been widely debated and many criticisms were based on its practicality, because a greater number of familiarization sessions have been needed for the elderly than in young adults [4,11,12,13,14,15,16,17]. Moreover, the performance of 1RM in older people is supposedly hindered by the need of high level of learning required [15], a high risk of injury [16] and the low reproducibility [17]. Taken together, these limitations lead to the suggestion for using predictive equations or submaximal tests to assess strength, which supposedly provides greater safety and less variability of the results in different exercises tested [18]. On the other hand, some investigations advocate the use of the 1-RM test in the elderly, indicating that the time of familiarization to the test may be similar to that of young adults [17], that the test is reproducible even in the untrained elderly [16] and that it has a low incidence of injuries [12]. These divergences can result from the different periods of familiarization, the heterogeneity of the groups and the inclusion of individuals with physical limitations in the sample.

Our hypothesis is that age and training level are not dependent factors for reproducibility of the maximal strength test in elderly women. Thus, this study aimed to verify the maximum muscle strength of elderly women and reproducibility of the test both before and after a 12-week training period. For this, we used the bench press and the 45° leg press exercises to perform 1-RM tests and re-tests, taking into consideration the training experience and strength level of the women.

## 2. Materials and Methods

### 2.1. Participants

Data regarding the training program as well as specific outcomes are described in another study involving the same participants [19]. Elderly women aged from 60 to 80 years were recruited through social networks. The participants were described in a study reporting the effects of training in functional outcomes. In summary, the sample comprised 376 women with a mean and standard deviation age of 68.5 ± 14.1 years, height of 162.7 ± 5.4 cm, body mass of 71.2 ± 15.9 kg, waist-hip ratio (WHR) of 0.85 ± 0.02 and body mass index (BMI) of 27.8 ± 4.7. The inclusion criteria used was not to have performed RT for at least three months before the data collection period, and to not have health issues that could preclude strength training, as cardiovascular, neurological or motor issues. None of the subjects were on any type of controlled or restricted diet and were advised to maintain their usual dietary patterns. The study followed the resolution of the National Health Council No. 466/12 of ethics in research involving human beings. Participants were informed about possible risks and discomforts that the test could cause. All participants signed a document stating their consent to participate in the study and health history containing information prior to the data collection period.

### 2.2. Designing

The experiment was carried out in two phases.

PHASE 1 (Pre-strength training): During the first week of the experiment, two training sessions were performed [13], separated by 48 h, with about 15 repetitions and self-selected load, as a familiarization strategy prior to the 1-RM test [20]. This procedure had the objective of reducing the error attempts on the day of tests [21]. After the first week, the 1-RM test was performed and retested after 48 and 72 h.

PHASE 2 (Post-strength training): After the 12-week period, during which the elderly women underwent a RT program (for more information see reference [19]), they underwent a new 1-RM test and retest with 48 to 72 h between them.

### 2.3. Procedures

#### 2.3.1. 1-RM Tests

##### Bench Press (BP)

The BP 1-RM test was performed with a barbell (180 cm; 9 kg) on a horizontal bench press (High On, Righetto Fitness, São Paulo, SP, Brazil). On the first day of the tests, participants performed a specific BP warm-up, which consisted of eight repetitions with a load between 40% and 50% of a perceived 1-RM load. After a one-minute recovery interval, participants performed a further warm-up set of six repetitions, with the load adjusted to 50–60% of a perceived 1-RM load. Thereafter, each participant had up to three attempts to reach the maximum load on the 1-RM test. If a repetition was completed successfully, the load increased between 0.5 and 10.0 kg and after five minutes a new attempt was made. Likewise, if a participant could not perform a repetition with the previously estimated load, the load was decreased between 0.5 and 10 kg, and after five minutes of rest a new attempt was made. However, this procedure was not necessary in any of the cases. During 1-RM testing the range of motion was controlled and participants had to touch the bar at the chest at the end of the eccentric phase and return to the initial position, with elbows fully extended at the end of the concentric phase. In addition, their neck, head, shoulders and hips were kept in contact with the bench and feet stayed in contact with the floor throughout the exercise. The speed of movement was self-selected by volunteers. Participants received verbal encouragement throughout the tests, and the same group of researchers performed all procedures. Between 48 and 72 h after the tests of phases 1 and 2, the retests were carried out following the same procedures [11].

##### Leg Press 45° (LP)

The test was performed in a LP machine that was declined at 45° (High On, Righetto Fitness, São Paulo, SP, Brazil). On the first day of tests, participants performed a specific warm-up in the same pattern previously described for the bench press. The range of motion was controlled with goniometer, in which participants flexed their knees at 100° (0° means full extension) at the end of the eccentric phase and returned to an initial position with the knees fully extended at the end of the concentric phase, avoiding hyperextension. The speed of movement was also self-selected by volunteers [11].

### 2.4. Statistical Analysis

After Shapiro-Wilk’s test for normality, data is presented as mean ± standard deviation. For comparisons between moments (before to after training) the Student t test was applied. For correlations the Pearson coefficient was used. Effect sizes (ES) was estimated by Cohen’s d and classified as small (0.20–0.39), medium (0.40–0.79) or large (≥0.80). For reproducibility Cohen’s Kappa Index was applied and strength of agreement was classified as weak (<0.20), reasonable (0.21–0.40), moderate (0.41–0.60), strong (0.61–0.80) and almost perfect (≥0.80) [22]. In addition, the mean coefficient of variation (CV) from individual test-retest CVs, intraclass correlation coefficient (ICC), standard error of measure [SEM = dp (√1 − ICC)] and minimal detectable change [MDC = SEM × (√2)] were presented. For all data analysis the SPSS (version 20.0, SPSS Inc., Chicago, IL, USA) was used. Statistical significance level was set at 5%.

## 3. Results

Descriptive and reproducibility results are presented in Table 1. In phase 1 (before strength training), relative 1RM strength was 0.19 kg/kgBM and 0.15 kg/kgBM for LP and BP, respectively. After 12 weeks of RT, in phase 2, the relative 1RM strength had a significant increase (LP, 0.59 kg/kgBM, *p* < 0.001, ES 0.96; BP, 0.31 kg/kgBM, *p* < 0.001, ES 0.86). Similarly, after 12 weeks of RT, in phase 2, the absolute 1-RM load showed significant improvements for LP (*p* < 0.001; ES = 0.97) and BP (*p* < 0.001; ES = 0.84) as previously reported [19].

Regarding the test-retest reproducibility, Kappa indices indicate values classified as “almost perfect” reliability for BP and LP 1RM, at both before and after training (Table 1). Similarly, the ICC for BP and LP 1RM was nearly perfect (0.99), regardless of the training status and maximal strength values of elderly women (Table 1). Additionally, regarding agreement, the MDC indicate that changes of 0.4 kg can be detected by the LP and the BP tests, which means detectable changes of 1.1% and 1.9%, respectively (Table 1).

**Figure 1:**
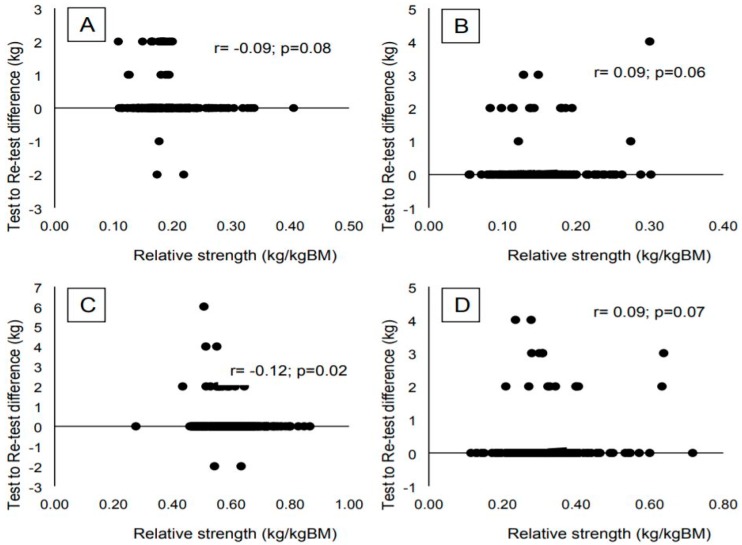
Correlations between strength level relative to body mass for LP (Panels **A** and **C**) and BP (Panels **B** and **D**) and differences between 1 RM test and re-test for before (Panels **A** and **B**) and after (Panels **C** and **D**) strength training intervention.

Figure 1 shows correlations for differences between tests and re-tests and relative strength for LP and BP. Despite significant or nearly entire significance, all situations presented very poor correlations.

## 4. Discussion

The present study aimed to verify the reproducibility of the 1-RM test in elderly women before and after a strength trained period, as well as the effects of aspects such as the age, anthropometrics, training experience and strength level of the women. In this sense the main findings are that the 1-RM testing is highly reproducible in elderly women regardless of training status or maximum strength (ICC > 0.99 for both LP and BP, before and after training). However, MDC seems to be slightly better after training periods detecting changes between 1 and 1.8%, which strengthens suggestions that training status might influence the consistency of the results obtained by a 1RM test. It is important to note that there was no report of injury, adverse event or even mild discomfort in this study, which is in agreement with previous findings in large populations [23]. Considering that maximum strength correlates with longevity [24,25], its measurement in the clinical practice of RT in the elderly becomes fundamental. Indeed, the 1RM test seems to be an interesting alternative for the evaluation of this group because it is highly reproducible and has simple application [26].

Our results strengthen those presented by Levinger et al. [26] that found high reproducibility (ICC > 0.99) of the 1-RM test in 53 untrained middle-aged individuals (51.2 ± 0.9 years). Similarly, LeBrasseur et al. [17] found high reproducibility rates of the 1-RM test in both LP and BP in elderly subjects (ICC > 0.98), with similar values for young and elderly individuals with low mobility. It is important to note that similar reproducibility rates were reported despite differences in sample and exercise protocols between studies. LeBresseur et al. [17] investigated 31 elderly men, using pneumatic machines in which the BP was performed in the seated position and, for the LP, the maximum knee flexion amplitude was 90° [17]. On the other hand, the present investigation was performed with 376 elderly women, with a leg press machine and greater knee flexion (100°), while BP was performed with a barbell. We believe that these are strengths of our experimental design, given the gender specificity and greater proximity of the exercises to the reality of coaches and trainers at gyms.

Previously, Knutzen, Brilla and Caine [27] contraindicated the use 1-RM tests in the elderly. However, the study evaluated the reliability of six equations for prediction of the 1-RM values. All equations tested underestimated the 1-RM, but the authors recommended its use for elderly because of the easiness of use, since all the tests ended between 7 and 10 repetitions. However, such a result does not disqualify the 1-RM test and, in fact, the prediction equations were not as accurate as the 1-RM test. Following the same line, Braith et al. [28] suggested the use of prediction equations for the untrained elderly and the 1-RM test for trained elderly. Their argument discussed the risk of injury based on a previous study by Pollock et al. [16]. Notwithstanding this, injuries reported by the elderly in the study of Pollock came from prior pathologies, since injuries occurred even in the control group. In the present study, there were no cases of injury, adverse event or discomfort in any of the 376 participants during or after 1-RM tests and is in agreement with the findings of Shaw, McCully and Posner [12], who evaluated 83 elderly (65.8 ± 6.2 years) men and women with and without RT experience. The results suggested that the 1-RM test is safe for this population, as there was no case of injury in the experienced group and only 2.4% of injuries incidence in the non-experience. It is worth noting that in the study of Shaw, there were no familiarization period before testing, and that individuals who suffered injury continued the study after recovering, without presenting any complaint of pain or discomfort. In this context, and coupled with our findings, the 1-RM test seems to be a safe method for the verification of maximal strength in the trained and untrained elderly.

Similar to our findings, Rydwik et al. [29] compared the reliability of 1-RM testing between the trained and untrained elderly. Their results did not show significant differences between groups and both reached high levels of reliability (r = 0.97). Likewise, Levinger et al. [26] found high reliability (r > 0.99) in 1-RM tests in untrained individuals using only one familiarization session. On the other hand, Ritti-Dias et al. [30] found low reliability in the 1-RM test using BP (r = 0.18) in young individuals without RT experience, however, no familiarization sessions were performed. Taken together, these findings suggest a high reliability for the 1-RM test in trained and untrained individuals when familiarization sessions are performed.

To our knowledge, the present study is the largest (n = 376) ever conducted among studies of this nature, especially considering the gender specificity. Current findings may have key implications relevant for assessment of strength and, indirectly, health, quality of life, longevity, risk of falls, and frailty, given its association with maximal strength in the elderly. However, some limitations should be considered when interpreting our findings. First, although we conducted 1-RM testing for both major upper and lower-body muscles, future studies may be conducted with similar familiarization and safety guidelines in order to replicate current finding in other muscle groups, even if it may be reasonable to expect similar findings [31]. Second, even considering 1RM as a gold standard for strength measures, it would be of interest to test its validity against other strength tests, including kinematic, kinect and physiological parameters.

## 5. Conclusions

Our findings suggest that the 1-RM test has high reproducibility for the elderly regardless of training status and maximum strength values, for both upper and lower body muscle groups. This method has been shown to be safe for elderly women as long as the methodological and familiarization processes with the applied RT exercises are respected.

## Figures and Tables

**Table 1:** Descriptive and reproducibility data for 1-RM tests and re-tests before and after RT experience (n = 376).

Procedures		Test	Re-Test	CV (%)	K (*p*)	ICC (*p*)	SEM (kg)	MDC, kg (%)
Before Training Intervention	Leg Press 1-RM (kg)	13.1 ± 2.5	13.2 ± 2.5	<1	0.92 (<0.001)	0.99 (<0.002)	0.2	0.3 (2.3)
	Bench Press 1-RM (kg)	10.1 ± 2.2	10.1 ± 2.3	<1	0.95 (<0.001)	0.99 (<0.002)	0.2	0.3 (3.1)
After Training Intervention	Leg Press 1-RM (kg)	39.3 ± 3.8 #	39.4 ± 3.7 #	<1	0.93 (<0.001)	0.99 (<0.001)	0.3	0.4 (1.1)
	Bench Press 1-RM (kg)	20.9 ± 4.3 #	21.0 ± 4.4 #	<1	0.95 (<0.001)	0.99 (<0.001)	0.3	0.4 (1.8)

#: Different from before training intervention at test and re-test moments for the same exercise; ±: Standard deviation; CV: Coefficient of variation; K: Kappa index; ICC: Intraclass correlation coefficient; SEM: Standard error of measurement; MDC: Minimal detectable change.
